# Analysis and Radiometric Calibration for Backscatter Intensity of Hyperspectral LiDAR Caused by Incident Angle Effect

**DOI:** 10.3390/s21092960

**Published:** 2021-04-23

**Authors:** Wenxin Tian, Lingli Tang, Yuwei Chen, Ziyang Li, Jiajia Zhu, Changhui Jiang, Peilun Hu, Wenjing He, Haohao Wu, Miaomiao Pan, Jing Lu, Juha Hyyppä

**Affiliations:** 1 Key Laboratory of Quantitative Remote Sensing Information Technology, Aerospace Information Research Institute, Chinese Academy of Sciences (CAS), Beijing 100094, China; tianwx@aircas.ac.cn (W.T.); lltang@aoe.ac.cn (L.T.); zyli@aoe.ac.cn (Z.L.); jjzhu@aoe.ac.cn (J.Z.); hewenjing@aoe.ac.cn (W.H.); wuhh@aircas.ac.cn (H.W.); panmiaomiao@aoe.ac.cn (M.P.); lujing18@mails.ucas.ac.cn (J.L.); 2 University of Chinese Academy of Sciences, Beijing 100049, China; 3 Department of Photogrammetry and Remote Sensing, Finnish Geospatial Research Institute (FGI), FI-02430 Masala, Finland; changhui.jiang@nls.fi (C.J.); hupeilun_818@163.com (P.H.); juha.hyyppa@nls.fi (J.H.); 4 Department of Forest Sciences, University of Helsinki, FI-00014 Helsinki, Finland

**Keywords:** Hyperspectral LiDAR (HSL), incidence angle, backscatter intensity, radiometric calibration, Lambertian–Beckmann model

## Abstract

Hyperspectral LiDAR (HSL) is a new remote sensing detection method with high spatial and spectral information detection ability. In the process of laser scanning, the laser echo intensity is affected by many factors. Therefore, it is necessary to calibrate the backscatter intensity data of HSL. Laser incidence angle is one of the important factors that affect the backscatter intensity of the target. This paper studied the radiometric calibration method of incidence angle effect for HSL. The reflectance of natural surfaces can be simulated as a combination of specular reflection and diffuse reflection. The linear combination of the Lambertian model and Beckmann model provides a comprehensive theory that can be applied to various surface conditions, from glossy to rough surfaces. Therefore, an adaptive threshold radiometric calibration method (Lambertian–Beckmann model) is proposed to solve the problem caused by the incident angle effect. The relationship between backscatter intensity and incident angle of HSL is studied by combining theory with experiments, and the model successfully quantifies the difference between diffuse and specular reflectance coefficients. Compared with the Lambertian model, the proposed model has higher calibration accuracy, and the average improvement rate to the samples in this study was 22.67%. Compared with the results before calibration with the incidence angle of less than 70°, the average improvement rate of the Lambertian–Beckmann model was 62.26%. Moreover, we also found that the green leaves have an obvious specular reflection effect near 650–720 nm, which might be related to the inner microstructure of chlorophyll. The Lambertian–Beckmann model was more helpful to the calibration of leaves in the visible wavelength range. This is a meaningful and a breakthrough exploration for HSL.

## 1. Introduction

Hyperspectral LiDAR (HSL) is a new remote sensing method. It has high spatial and spectral information detection ability, which greatly compensates for the disadvantage of the traditional single-wavelength LiDAR in target attribute identification by offering a “colorful” point cloud. In addition to the geometric information, it can record the backscattered optical power of each scanned target at multiple wavelengths. The backscattered optical power is internally converted to a voltage, amplified in the system, and finally transformed into a Digital Number (DN), which is called “backscattered intensity”. [1,2,3]. The HSL system generates point clouds [x,y,z,I(λ)], and I(λ) denotes the intensity, which is a continuous function of laser wavelength, λ. The backscatter intensity of laser at different wavelengths has an accurate one-to-one correspondence with the three-dimensional coordinate information. It has the advantage of pixel-level fusion without registration, which avoids the additional complex data processing process caused by the fusion and registration of traditional single-wavelength LiDAR and hyperspectral remote sensing imaging data. At present, compared with the single-wavelength LiDAR system, the HSL system can be applied to fine classification and quantitative parameter inversion. For example, fine classification of tree species [4] and estimation of plant physiological information (leaf area density [5], leaf water content [6], nitrogen content [7], and chlorophyll content [8,9]).

The backscatter intensity of the HSL is associated with the reflectance properties of the targets. Targets of higher reflectance will reflect a more significant incident laser radiation, thereby increasing the backscattered intensity. Therefore, the backscatter intensity of different wavelengths is a substantial physical quantity reflecting targets’ surface characteristics. In the process of laser scanning, the backscatter intensity is affected by atmospheric transmission attenuation, system characteristics, target reflection characteristics, incidence angle, distance, and other factors [10,11]. Therefore, it is necessary to calibrate the backscatter intensity data of HSL for further post-processing. In a scanning experiment of HSL, the system parameters and atmospheric influence factors can be regarded as invariable when the HSL system performance is stable, and the experimental conditions remain unchanged. If the distance between the target and the HSL system remains unchanged, the main factors affecting the backscatter intensity of HSL are target reflection characteristics and incidence angle. For LiDAR, the incident angle is defined as the angle between the laser incident direction and the normal direction of the target surface. Therefore, a more comprehensive radiometric calibration method for incident angle effect is needed to eliminate the influence of incidence angle effect on the backscatter intensity of HSL.

At present, there are many studies on the intensity calibration of the traditional single-wavelength LiDAR system. These methods can be divided into four categories: physical model, empirical-driven model, calibration method based on reference target, and data-driven model. The physical model mainly uses the physical laws to explain the laser scattering process accurately. The empirical-driven model is mainly based on Bidirectional Reflectance Distribution Function (BRDF) models. For the distance and incidence angle effects, many scholars have proposed new calibration models such as the Oren-Nayar model [12,13], the Phong model [14,15], and the Beckmann model [6,16], etc. These models can be applied to Lambert and non-Lambert reflectors. The calibration method based on reference target is mainly based on natural targets [17] or a standard reference board with known reflectance to eliminate system effect, incidence angle effect, and distance effect. The data-driven model [18,19] directly establishes the mathematical model between the backscatter intensity and various influencing factors. By calculating the model parameters, the backscatter intensity data are corrected.

In addition, there are also some researches on radiometric calibration of HSL. These methods can be divided into three categories: the method based on reference target [20,21], the method based on spectral ratio [22], and the method based on the Lambertian model [23]. The reference target method mainly uses the standard reflectance board with known reflectance as the reference target. Under the same distance and incident angle, the reference target’s intensity is used to normalize the backscatter intensity of each wavelength of the measured target. This method has poor adaptability and is only suitable for simple application scenarios. The method based on spectral ratio uses the principle that the intensity ratio between two different wavelengths is independent of distance and incident angle. The reflection characteristics of different targets can be distinguished by spectral ratio indirectly. However, this method does not directly calculate the reflectance related to the target’s characteristics and is only suitable for the recognition of plants and a small part of the target. According to the method based on the Lambertian model, the backscatter intensity of LiDAR is proportional to the cosine of the incident angle. The Lambertian model can be used to correct the incident angle effect for the ideal Lambertian surface, which is widely used in many intensity calibration applications. However, there is almost no ideal Lambertian surface, and the characteristics of natural targets are very complex in practical applications. In general, there are both diffuse and specular reflections in the reflection of LiDAR. For some targets with glossy surfaces, the reflection characteristics deviate from the Lambertian model, obviously. Hot-spot effects caused by the specular reflections may appear and are detrimental for further applications. Therefore, it is not enough to use the Lambertian model to correct the incidence angle effect of backscatter intensity of HSL. The accuracy and adaptability of the existing calibration methods for angle effect still need to be improved.

In this study, we mainly discuss the radiation calibration method of the incident angle effect for the backscatter intensity of HSL. Considering the different characteristics of the target surface, we study the influence of the incident angle on the backscatter intensity of different wavelengths. We propose an adaptive threshold calibration model for the incident angle effect, which is used to retrieve the diffuse reflectance parameters and reflectance of targets. The research provides a comprehensive theory that can be applied to the target with characteristics from diffuse to specular reflections and can be further applied to target classification and quantitative parameter inversion. By considering the effects of diffuse reflections and specular reflections, the incidence angle effect can be accurately corrected. Compared with the Lambertian model, the proposed model has higher calibration accuracy. Compared with the Lambertian model, the average improvement rate to the samples in this study was 22.67%. Compared with the results before calibration with the incidence angle of less than 70°, the average improvement rate of the Lambertian–Beckmann model was 62.26%. The proposed procedure improves the usability of HSL and considerably mitigates the problems caused by the angle effect of HSL.

The rest of the study is organized as follows: The proposed method and experiment setup are illustrated in Section 2. Section 3 presents the results. Discussion and conclusions are provided in Section 4 and Section 5, respectively.

## 2. Materials and Methods

This section is divided into three subsections, and the first subsection is the HSL System design, components, and description. The second subsection presents the Radiometric Calibration Model of Incident Angle Effect. The third subsection presents the incidence angle experiments.

### 2.1. HSL System

**Figure 1:**
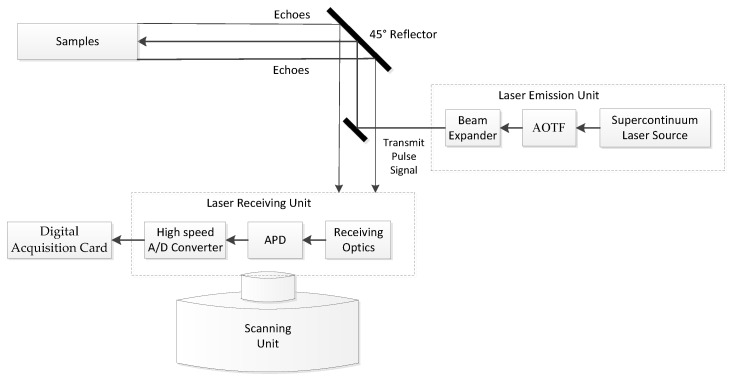
The optical layout of the HSL System.

The HSL System [20,24] consists of three units, including a laser emission unit, a full-waveform signal receiving unit, and a scanning control unit. Figure 1 presents the design of the HSL System.

The laser emission unit is mainly composed of a supercontinuum laser, acousto-optic tunable filter (AOTF), collimating beam expander, and mirror. The supercontinuum laser emits a broadband spectrum laser beam with a wide range of wavelengths (400–2500 nm). AOTF is capable of emitting different wavelength laser signals at different times. It provides a fast tuning speed (microsecond) and a wide wavelength range (430–1450 nm). The spectral resolution is 2–10 nm. The laser beam expander is mainly used to collimate the laser beam and change it into a parallel beam. The mirror’s main function is to transmit the tuned monochromatic laser to the detection target after 45° deflection.

The laser receiving unit is mainly composed of a receiving optical system, an Avalanche Photon Diode (APD) detector module, and a digital acquisition card. A Cassegrain telescope optical system is employed to collect the laser echo signals of multiple wavelengths from the targets. A silicon APD detector module is placed on the telescope’s focal point to collect and amplify the backscattered laser echoes and transform them into electronic signals. A computer controls the digital acquisition card to collect the full waveform echo signal triggered by a laser transmitting signal (10 GHz sampling rate). The distance is obtained by measuring the time difference between the triggering signal and the reflected signal from the target.

The laser scanning control unit is mainly composed of a two-dimensional turntable. It is used to synchronously control the transmitting beam of the laser emission unit and the receiving field of the laser receiving unit.

### 2.2. Radiometric Calibration Model of Incident Angle Effect

#### 2.2.1. Lidar Equation

The laser system emits laser beams to the target and records the returned laser pulse signals after being backscattered from the target. This process can be expressed by the Lidar Equation as Equation (1) [25]. It can be regarded as the product of the four factors of atmosphere, target characteristics, optical system, and laser receiver. It provides a quantitative theoretical basis for laser intensity radiometric calibration.

(1) $$P_{r}=\frac{P_{t}D_{r}^{2}}{4\pi R^{4}\beta_{t}^{2}}\sigma \eta_{atm}\eta_{sys}$$

where $P_{r}$ is the total received power of the sensor, $P_{t}$ is the transmitted laser power, *R* is the distance between the target and the sensor, $\beta_{t}$ is the transmitter beam divergence angle ($\beta_{t}$ is very small, $\beta_{t}\to 0,\;\sin\beta_{t}\approx \beta_{t}$), $D_{r}$ is the receiver aperture diameter of the receiving optics, $\eta_{atm}$ is the influence factor of the signal transmission in the atmosphere, $\eta_{sys}$ is the parameter of the LiDAR system, and σ is the backscatter cross-section of the target and is related to the reflection characteristics of the target. Suppose that θ (laser incidence angle) is the angle between the incident laser beam and the surface normal vector, Ω is the solid angle in the backscatter direction of the LiDAR, $A_{s}=\frac{\pi R^{2}sin\beta_{t}^{2}}{4}\approx \frac{\pi R^{2}\beta_{t}^{2}}{4}$ is the target effective receiving area, and ρ is the reflectance of the target. The backscatter cross-section of the target, σ, is expressed as:

(2) $$\sigma =\frac{4\pi \rho }{\Omega }A_{s}\cos\theta$$

The formula shows that the backscatter characteristic of the target depends on the size of the spot on the target, the reflectance, and the scattering directivity. When the laser beam irradiates the ideal Lambertian surface, only diffuse reflection occurs. When the incident angle is fixed, the reflected laser beam of the target is evenly scattered to a hemisphere. In this case, Ω=π, the backscatter intensity is a constant when viewed from any angle. For the Lambertian surface, the light intensity received at different angles is proportional to the cosine of the incident angle, θ:

(3) $$\sigma =\pi R^{2}\beta_{t}^{2}\rho \cos\theta$$

Substituting Equation (3) into Equation (1), the Lidar Equation in case of diffuse reflection is obtained as follows:

(4) $$P_{r}=\frac{P_{t}D_{r}^{2}\rho \cos\theta }{4R^{2}}\eta_{atm}\eta_{sys}$$

Based on the Lidar Equation, the received power of different wavelength of HSL can be written as:

(5) $$P_{r}(\lambda )=\frac{P_{t}(\lambda )D_{r}^{2}\rho (\lambda )\cos\theta }{4R^{2}}\eta_{atm}(\lambda )\eta_{sys}(\lambda )$$

where $P_{r}\left(\lambda \right)$ is the total received power of the sensor at a certain laser wavelength, λ, of the HSL system. Suppose that the object is an ideal Lambertian surface and the atmospheric condition is constant. $P_{t}\left(\lambda \right),D_{r},\eta_{atm}\left(\lambda \right)$ and $\eta_{sys}\left(\lambda \right)$ can be considered as four constants during a laser scanning process. Equation (5) can be simplified as:

(6) $$P_{r}(\lambda )=\frac{C(\lambda )\rho (\lambda )\cos\theta }{R^{2}}$$

(7) $$C(\lambda )=\frac{P_{t}(\lambda )D_{r}^{2}}{4}\eta_{atm}(\lambda )\eta_{sys}(\lambda )$$

where *C* depends on the system and the atmospheric effects, as in Equation (7). Generally, the backscatter intensity, I(λ), of HSL is positively correlated with the received power, $P_{r}\left(\lambda \right)$ [20]. Therefore, Equation (6) can be expressed as Equation (8):

(8) $$I(\lambda )\propto \frac{C(\lambda )\rho (\lambda )\cos\theta }{R^{2}}$$

where ∝ represents a positive correlation. Therefore, for the ideal Lambertian surface, the backscatter intensity of different wavelengths of HSL is directly proportional to cosθ and inversely proportional to $R^{2}$. This is the Lambertian model. The backscatter intensity in the normal direction, $f_{0}\left(\lambda \right)$, is:

(9) $$f_{0}(\lambda )=\frac{C_{1}(\lambda )\rho (\lambda )}{R^{2}}$$

where $C_{1}(\lambda )$ is a function of λ and depends on the system and the atmospheric effects. Assuming that the distance is constant during the laser scanning process, the backscatter intensity of different incidence angles is corrected to the normal direction based on Equation (10), where $I{\left(\theta ,\lambda \right)}_{s}$ is the corrected intensity to the Lambertian model.

(10) $$I{\left(\theta ,\lambda \right)}_{S}=f_{0}(\lambda )\cos\theta$$

#### 2.2.2. Lambertian–Beckmann Model

There is almost no ideal Lambertian surface in many applications, and the reflection characteristics of the scanning target are often complex. The target surface is composed of many micro planes with different orientations. Due to the surface roughness or specular reflection of the scanning target surface, the reflection characteristics deviate from the Lambertian model. For the targets with glossy surfaces, the specular reflection will increase the backscatter echo power. Therefore, it is not sufficient to correct the backscatter intensity of HSL caused by the incident angle effect using the Lambertian model and Equation (10).

**Figure 2:**
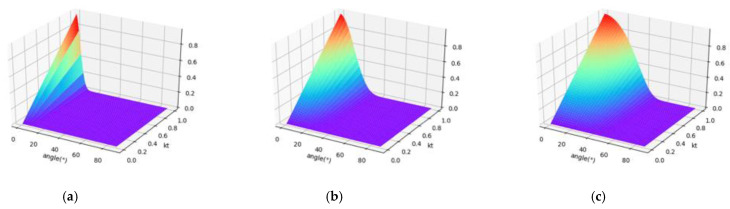
The variation of the specular reflection intensity, $I{\left(\theta ,\lambda \right)}_{t}$, with the incident angle, *θ*, and $k_{d}\left(\lambda \right)$. Parameter m is fixed at 0.1, 0.3, and 0.5, respectively. (**a**) m = 0.1; (**b**) m = 0.3; (**c**) m = 0.5.

Beckmann and Spizzichino proposed the Beckmann Model [26] in 1987 to model the intensity characteristics of specular reflection.

(11) $$I{\left(\theta ,\lambda \right)}_{t}=f_{0}(\lambda )k_{t}(\lambda )\cdot \frac{e^{-\frac{\tan^{2}\theta }{m^{2}}}}{\cos^{5}\theta }$$

where $I{\left(\theta ,\lambda \right)}_{t}$ is the specular reflection intensity at the wavelength, *λ*, and the incident angle, θ, $f_{0}\left(\lambda \right)$, is the backscatter intensity in the normal direction; $k_{t}\left(\lambda \right)$ is the coefficient of the specular reflection component, which is between 0 and 1 and related to the surface smoothness of the target. The smaller $k_{t}\left(\lambda \right)$ is, the rougher the surface is, and the reflection is emission. The larger $k_{t}\left(\lambda \right)$ is, the smoother the surface is and the higher the degree of light convergence is. Parameter *m* is the surface roughness, which is expressed by the standard deviation of height distribution. Parameter m explains the influence of surface roughness on the scattering of LiDAR in the specular reflection direction. The value is between 0 (smooth surface) and 0.6 (rough surface). Figure 2 shows the variation of the specular reflection intensity, $I{\left(\theta ,\lambda \right)}_{t}$, with the incident angle, *θ*, and $k_{t}\left(\lambda \right)$. 

In Equation (10), the coefficient of the diffuse reflection component is 1. In practice, specular reflection and diffuse reflection exist simultaneously, and the degree of them is different for different targets. The natural surface is simulated as a combination of specular reflection and diffuse reflection. The linear combination of the Lambertian model and Beckmann model provides a comprehensive theory that can be applied to various surface conditions from glossy to rough to simulate the backscatter intensity. Assuming that the coefficient of diffuse reflection component is $k_{d}\left(\lambda \right)$, $k_{d}\left(\lambda \right)+k_{t}\left(\lambda \right)=1$. Therefore, Equation (10) can be written as:

(12) $$I{\left(\theta ,\lambda \right)}_{s}=f_{0}(\lambda )k_{d}(\lambda )\cos\theta$$

The Lambertian–Beckmann model is expressed as Equation (13):

(13) $$I(\theta ,\lambda )=f_{0}(\lambda )\left[k_{d}(\lambda )\cos\theta +(1-k_{d}(\lambda ))\cdot \frac{e^{-\frac{\tan^{2}\theta }{m^{2}}}}{\cos^{5}\theta }\right]$$

**Figure 3:**
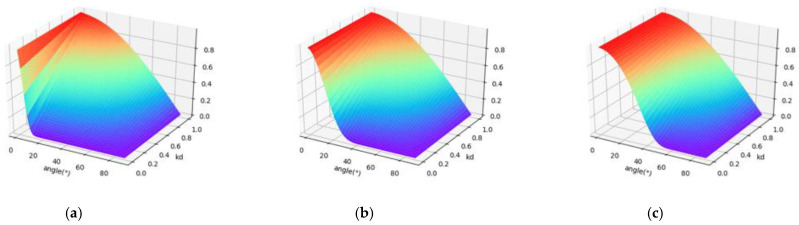
The variation of the backscatter intensity, I(θ,λ), with the incident angle, *θ*, and $k_{d}\left(\lambda \right)$. Parameter m is fixed at 0.1, 0.3, and 0.5, respectively. (**a**) m = 0.1; (**b**) m = 0.3; (c) m = 0.5.

I(θ,λ) is the backscatter intensity of the HSL at the wavelength, *λ*, and at the incident angle, θ. Figure 3 shows the variation of the backscatter intensity, I(θ,λ), with the incident angle, θ, and $k_{d}\left(\lambda \right)$.

#### 2.2.3. Radiometric Calibration Model

Figure 2 and Figure 3 show that when the laser beam irradiates the ideal Lambert surface, only diffuse reflection occurs; ($k_{d}\left(\lambda \right)$ = 1, $k_{t}\left(\lambda \right)$ = 0). The target surface has the same backscatter intensity in all directions, and the backscatter intensity conforms to the Lambertian model. When the laser beam irradiates the ideal specular surface ($k_{d}\left(\lambda \right)$ = 0, $k_{t}\left(\lambda \right)$ = 1, m = 0), the reflected laser and incident laser are symmetrically distributed on both sides of the normal direction of the surface, and the reflection angle is equal to the incident angle according to the law of light reflection. For HSL, the position of laser emission and laser reception is the same. For a generally glossy surface, the surface is composed of many micro planes with different orientations, and the spatial distribution of specular reflection intensity has certain directionality. The higher specular intensity is distributed around the direction of specular reflection. The smaller the incidence angle is, the higher the specular reflection intensity received by the sensor is. The larger the incidence angle is, the smaller the specular reflection effect is, and the weaker the specular reflection intensity. When the incident angle exceeds a certain angle threshold, the specular intensity is 0. The angle threshold increases with the increase of roughness parameter, *m*.

Therefore, the threshold of incidence angle is assumed to be $\theta_{T}$. When the incident angle is larger than $\theta_{T}$, although the specular light exists, the sensor cannot receive the specular laser. In this case, only the diffuse laser reaches the sensor, and the specular effect can be ignored. When the incident angle is less than $\theta_{T}$, the sensor can receive part of the specular light and diffuse light at the same time. Therefore, the Lambertian–Beckmann model is improved to a piecewise equation:

(14) $$\{\begin{array}{ll} I(\theta ,\lambda )=f_{0}(\lambda )\left[k_{d}(\lambda )\cos\theta +(1-k_{d}(\lambda ))\cdot \frac{e^{-\frac{\tan^{2}\theta }{m^{2}}}}{\cos^{5}\theta }\right] & \theta <\theta_{T}\\ I(\theta ,\lambda )=f_{0}(\lambda )k_{d}(\lambda )\cos\theta & \theta \geq \theta_{T} \end{array}$$

where the threshold of incidence angle, $\theta_{T}$, is calculated as follows: with the increase of the incidence angle, the critical angle is the threshold, $\theta_{T}$, when the specular reflection component, $I{\left(\theta ,\lambda \right)}_{t}$, tends to zero.

In theory, the reflection characteristics of all targets are the combination of diffuse reflection and specular reflection. In the process of backscatter intensity radiometric calibration, the influence of diffuse reflection and specular reflection should be considered simultaneously. Based on the fitted model parameters, an adaptive threshold radiometric calibration method is designed as Equation (15):

(15) $$\{\begin{array}{ll} I{\left(\theta ,\lambda \right)}_{c}=\left[I(\theta ,\lambda )-f_{0}(\lambda )\cdot (1-k_{d}(\lambda ))\cdot \frac{e^{-\frac{\tan^{2}\theta }{m^{2}}}}{\cos^{5}\theta }\right]\cdot \frac{\cos\theta_{0}\cos\theta_{s}}{\cos\theta } & \theta <\theta_{T}\\ I{\left(\theta ,\lambda \right)}_{c}=I(\theta ,\lambda )\frac{\cos\theta_{s}}{\cos\theta } & \theta \geq \theta_{T} \end{array}$$

where $\theta_{s}$ is the standard incidence angle, $\theta_{0}$ is the incidence angle in the normal direction, $I{\left(\theta ,\lambda \right)}_{c}$ is the corrected intensity, and I(θ,λ) is the original backscatter intensity.

### 2.3. Incidence Angle Experiments

In order to study the effect of incidence angle on the laser backscatter intensity of HSL, the experiments were performed under a controlled laboratory condition, and test samples with a wide range of variance of surface roughness were selected purposely. The experimental samples mainly include (Table 1): three standard reference targets with different reflectance (99%, 70%, 40%); wood product; sidewalk brick; white floor tiles (ceramics); marble tiles; a car shell sample; and six kinds of common plant leaf samples in Beijing (Including Fraxinus pennsylvanica, yellow leaf of Fraxinus pennsylvanica, Eucommia ulmoides, Magnolia denudate, Ficus elastic, and Codiaeum variegatum).

**Figure 4:**
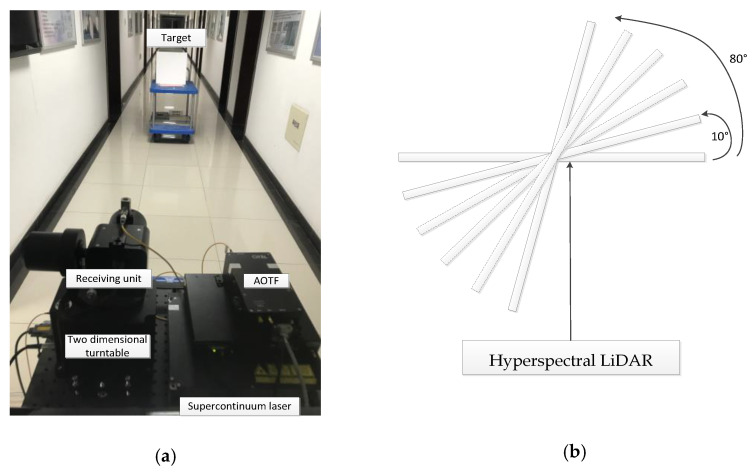
Measuring platform: (**a**) The HSL experimental platform; (**b**) Sample angle measuring platform.

A simple angle measuring platform was established to measure the incident angle of backscatter intensity (Figure 4). A protractor, which can be rotated horizontally to change the incidence angle, was fixed on the platform to display the incidence angle. The samples were measured at different incident angles and at a fixed distance (approximate 4 m). The incident angle varied between 0° and 80° with the range of 10° as the incremental step. The 99% standard reference board with the same scanning distance was used to calculate the reflectance. The backscatter intensity was recorded at 26 wavelength channels (650~900 nm with 10 nm step) for all test samples. We filtered out the signals less than 650 nm for their low energy and beyond 900 nm for their rapid decrease in transmitting energy density for better signal/noise ratio with collected echo signal. In the measurement process, the leaves were fixed on a 60 cm × 60 cm black back-plate with low reflectance (5%) in the whole wavelength to mitigate the influence of the background.

The whole experiment was divided into separate times because each sample took a long time. Experiments of the same sample were carried out at the same time to keep their HSL energy at a constant level. We recorded 30 backscatter intensities for each wavelength and each incident angle. We took the arithmetic mean of 30 intensities as the backscatter intensity of the sample in each incident angle to increase the final signal/noise ratio.

In order to compare the reflectance obtained by active and passive methods and verify the spectral analysis ability of HSL, we compared the spectral reflectance measured by HSL (active method) with that measured by a Spectra Vista Corporation (SVC) spectrometer (passive method) in the corresponding wavelength range at the angle of normal incidence. The results of the HSL and SVC spectrometer were compared and used for spectral analysis.

## 3. Results

To verify the spectral analysis ability of HSL, we compared the spectral reflectance measured by HSL with that measured by the SVC spectrometer in the corresponding wavelength range in Section 3.1. Section 3.2 mainly analyzes the incidence angle effect at different wavelengths measured by the HSL for the samples. The Lambertian–Beckmann model proposed in this paper is used to correct the backscatter intensity of HSL caused by the incidence angle effect in Section 3.3.

### 3.1. HSL and SVC Spectrometer Measurements

The SVC spectrometer measured the reflectance in clear weather and natural sunlight. Before each measurement, the 99% standard reflectance board was measured with an SVC spectrometer as the reference. Then, different samples were measured, and every measurement was made on different parts of each sample. The samples’ reflectance was obtained by the ratio of the reflected signal value of the samples to the reflected signal value of the standard reflectance board. Five groups of reflectance data were measured for each sample, and the arithmetic mean value was calculated as the reflectance measured by the SVC spectrometer.

After the measurement, the samples were taken back to the laboratory for reflectance measurement using HSL. We measured the response voltage of the samples and that of the standard reflectance board at the same position. When the laser signal was emitted perpendicularly, we used the following calibration model as Equation (16) to determine the samples’ reflectance [20].

(16) $$\rho_{obj}(\lambda )=\frac{f_{APD\_obj}(\lambda )}{f_{APD\_ref}(\lambda )}\rho_{ref}(\lambda )$$

where $f_{APD\_ref}(\lambda )$ is the backscatter intensity value of the standard reflectance board, $f_{APD\_obj}(\lambda )$ is that of the detection target, $\rho_{ref}(\lambda )$ is the reflectance of the standard reflectance board, and $\rho_{obj}(\lambda )$ is the reflectance of the detection target.

**Figure 5:**
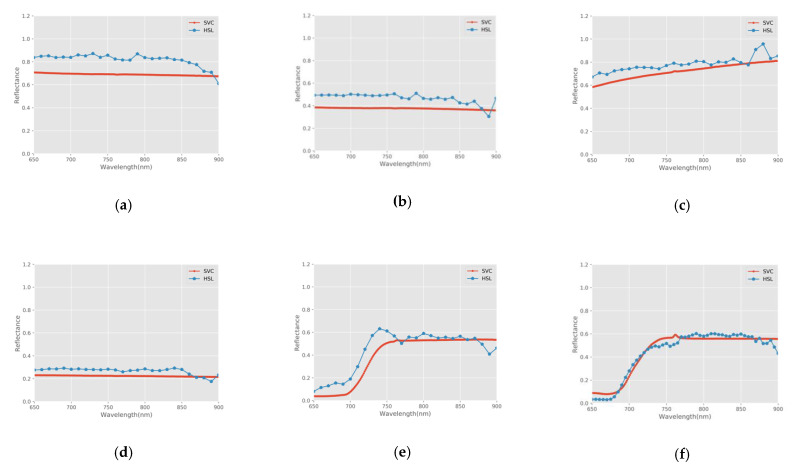

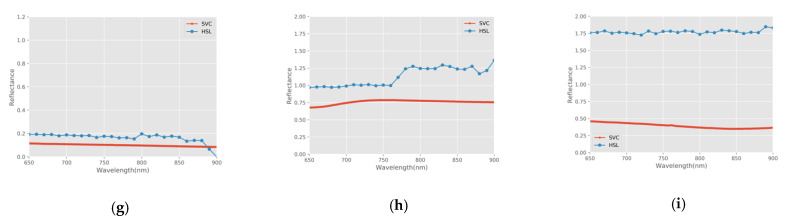
Reflectance comparison of HSL and SVC spectrometer. (**a**) 70% standard reference board; (**b**) 40% standard reference board; (**c**) Wood product; (**d**) Sidewalk brick; (**e**) Ficus elastic; (**f**) Codiaeum variegatum; (**g**) Marble tile; (**h**) Floor tile; (**i**) Car shell sample.

We computed the distribution curves of the reflectance of samples from the HSL and SVC spectrometer. Because floor tile and the car shell sample have obvious specular reflection characteristics, the reflectance measured by HSL is greater than 1, which is far greater than that measured by SVC. In this paper, Ficus elastic and Codiaeum variegatum were selected to show the measurement results of leaf samples. Figure 5 shows the reflectance comparison for several representative samples, including 70% standard reference board, 40% standard reference board, wood product, sidewalk brick, marble tile, floor tile, car shell sample, Ficus elastic, and Codiaeum variegatum.

Figure 5 shows that the extracted reflectance of 70% standard reference board, 40% standard reference board, wood product, sidewalk brick, Ficus elastic, Codiaeum variegatum, and marble tile obtained by HSL is consistent with that obtained by the SVC spectrometer, and the shapes were similar. The reflectance of the floor tile and the car shell sample measured by HSL were much higher than those measured by SVC and were higher than 1, which is not consistent with the physical principles. This was consistent with the specular reflection characteristics of the two samples, and it was a hot-spot effect caused by the specular reflections. The spectral curve of green leaves is low in the visible region and high in the near-infrared region, with obvious red edges, which conforms to the spectral characteristics of leaves. For the other samples, the reflectance measured by HSL was slightly higher than that by SVC, partly because the transmitting laser energy was stronger. We also found that the reflectance curve has obvious fluctuation near 900 nm because there is a poor signal/noise ratio. The results demonstrated the excellent fitness of the measurements from the two devices. It can be preliminarily concluded that the reflectance results of the HSL system are reliable.

### 3.2. Incidence Angle Effect of Backscatter Intensity

**Figure 6:**
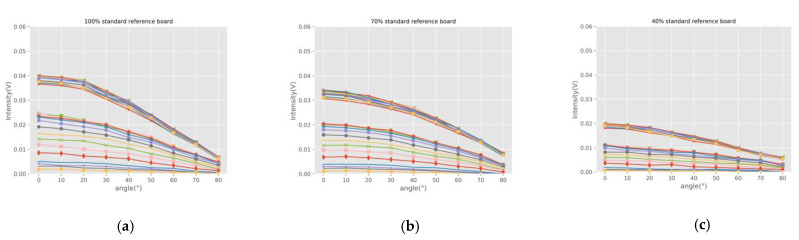

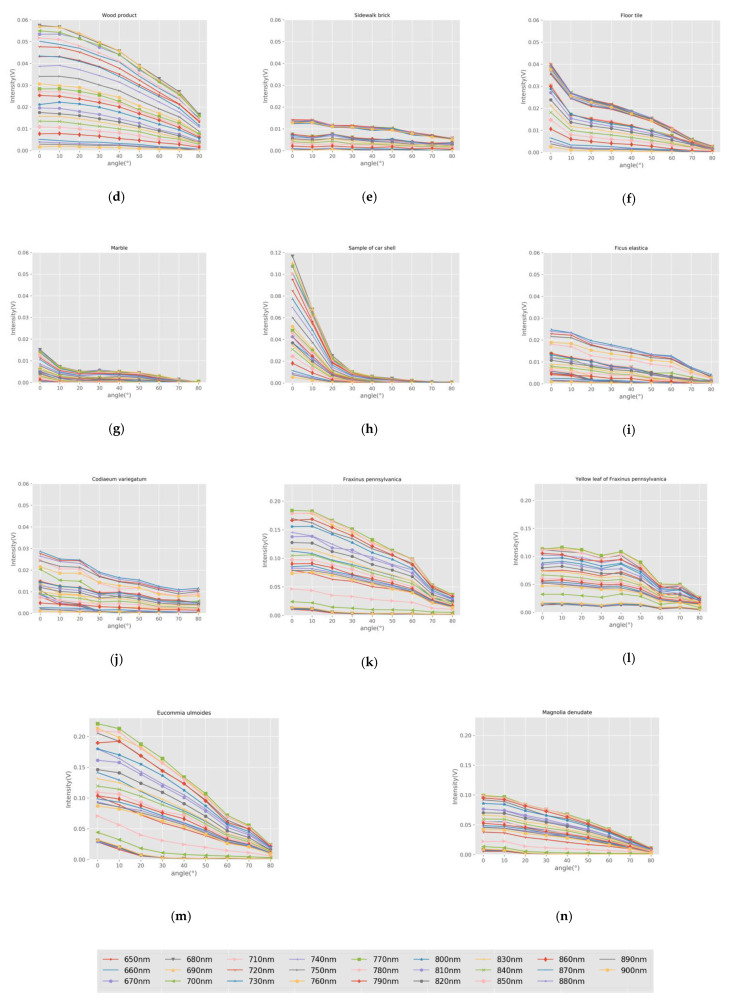
Incidence angle effect at different wavelengths measured with the HSL. (**a**) 100% standard reference board; (**b**) 70% standard reference board; (**c**) 40% standard reference board; (**d**) Wood product; (**e**) Sidewalk brick; (**f**) Floor tile; (**g**) Marble tile; (**h**) Car shell sample; (**i**) Ficus elastic; (**j**) Codiaeum variegatum; (**k**) Fraxinus pennsylvanica; (**l**) Yellow leaf of Fraxinus pennsylvanica; (**m**) Eucommia ulmoides; (**n**) Magnolia denudate.

In order to analyze the incident angle effect of samples with different reflection characteristics, we drew the curves of backscatter intensity, varying with the angle. Figure 6 shows the incidence angle effect at different wavelengths measured with the HSL for the samples. The intensity curves of twenty-six wavelengths present similar features. As can be seen from the whole, the backscatter intensity decreases as the incidence angle increases for a fixed distance sample. For different samples at the same incidence angle, the intensity is proportional to the reflectance. It shows that the incidence angle significantly affects the originally recorded backscatter intensity.

Based on Equation (8), the backscatter intensity of HSL is directly proportional to cos θ at a fixed distance for the Lambertian sample. In Figure 6, we can see that the intensity curves of standard reference boards, wood product, sidewalk brick, and leaves approximately conform to Equation (8). Samples of floor tile, marble tile, and car shell have different degrees of specular reflection characteristics. Regarding samples with specular reflection characteristics, the smaller the incidence angle is, the higher the specular reflection intensity received by the sensor is. With the increase of incident angle, the specular reflection effect decreases rapidly. Therefore, the intensity curves of these samples decrease rapidly when the angle is small, and the law is similar to that of the Lambert samples when the angle is large. This is especially obvious for the car shell sample. This phenomenon is consistent with the hot-spot effect in Figure 5h,i, measured in the incident angle of 0°.

The experiments were divided into different times, and the AOTF had two input interfaces. The output wavelength range of the first interface is 430 nm–770 nm, and the other is 770 nm–1450nm. If the interface is replaced during the experiment, the output energy of AOTF cannot guarantee its repeatability and normally has minor instability. Therefore, some of the samples, such as the standard reference board, have lower backscatter intensity when the wavelength is greater than 770 nm. However, experiments of the same sample were carried out in the same experiment, and this phenomenon does not affect the calculation of reflectance and the analysis of angle effect in this study.

### 3.3. Backscatter Intensity Calibration Based on Lambertian Beckmann Model

**Figure 7:**
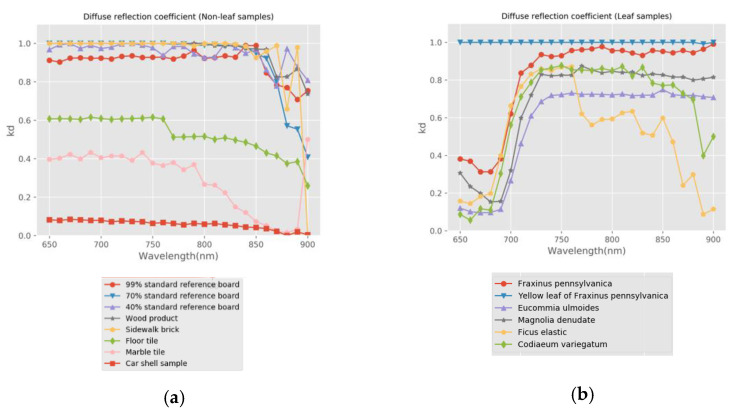
The relationship between diffuse reflection coefficient, $k_{d}$, and wavelength, λ. (**a**) Non-leaf samples; (**b**) Leaf samples.

**Figure 8:**
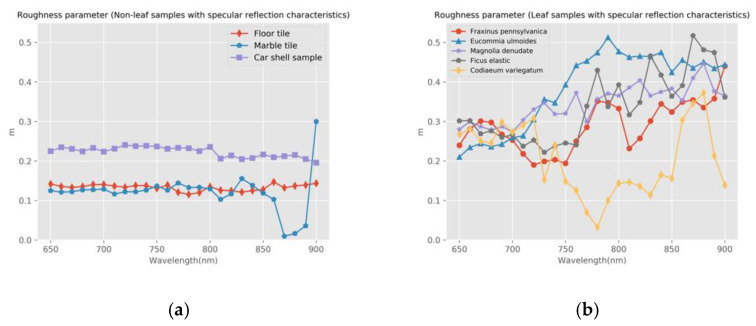
The relationship between roughness parameter, m, and wavelength, λ. (**a**) Non-leaf samples; (**b**) Leaf samples.

The Lambertian–Beckmann model was used to simulate the backscatter intensity of HSL. We performed nonlinear curve fitting for each wavelength and each sample with the piecewise Lambertian–Beckmann model in Equation (14) and used the least square method. The software and the procedure of parameters estimation were based on Python. The values of the $k_{d}\left(\lambda \right)$, *m*, and $\theta_{T}$. parameters in Equation (14) were obtained. The relationships between the sample’s diffuse reflection coefficient, $k_{d}\left(\lambda \right)$, roughness parameter, *m*, and different wavelengths are shown in Figure 7 and Figure 8. The parameter m is only calculated for the samples with specular reflection characteristics. The relationship between $\theta_{T}$ and different wavelengths are shown in Table 2.

After analyzing the calculation results of model parameters, it can be concluded that:

1. For the three standard reflection boards, sidewalk brick, and the wood product in this experiment, the coefficient $k_{d}\left(\lambda \right)$ is close to 1, and the angle threshold $\theta_{T}$ is 0°, indicating that there is almost no specular reflection in these samples.
2. The floor tile, the car shell sample, and the marble tile in this experiment are samples with obvious specular reflection characteristics. The diffuse reflection coefficient, $k_{d}$, of floor tiles is about 0.52, and the roughness coefficient, m, is about 0.15. The diffuse reflection coefficient, $k_{d}$, of the car shell sample is about 0.1, and the roughness coefficient, m, is about 0.21. The diffuse reflection coefficient, $k_{d}$, of marble tile is about 0.4, and the roughness coefficient, m, is about 0.12. This result is consistent with the optical reflection characteristics of the sample itself. The specular reflection of the car shell sample is the most obvious, and it is consistent with Figure 6h.
3. For the leaf samples in this experiment, the coefficient, $k_{d}$, shows a significant trough in the range of visible wavelength and increases in the range of near-infrared wavelength. This trend is consistent with the red edge effect of leaves. This indicates that the leaves have a stronger specular reflection in the visible wavelength range. Roughness parameter m shows a relatively stable trend and represents the overall roughness of this target. For the yellow leaf of Fraxinus pennsylvanica, the diffuse reflection coefficient, $k_{d}$, is close to 1 and remains unchanged. Therefore, the yellow leaf of Fraxinus pennsylvanica is closest to the Lambertian model.
4. The backscatter intensity of the glossy green leaf surface is higher than that of the matte or yellow leaf surface, especially near the normal direction. Figure 7b shows that Eucommia ulmoides has the lowest diffuse reflection coefficient and the highest specular backscatter intensity in all leaf samples. Combined with Figure 6m, it can be seen that the backscatter intensity of Eucommia ulmoides drops sharply between 0° and 20°.
5. For non-leaf samples in this experiment, the specular reflection effect is similar in different wavelengths. For the leaf samples in the experiment, the differences of specular reflection effect and incident angle effect were different, and the specular reflection effect was larger in the visible region for waxy or glossy leaves. For rough and dull leaves, the effect of specular reflection is small or negligible. Therefore, the impact of the incident angle effect on different wavelengths is different. In the process of radiometric calibration, the angle effect in different wavelengths needs to be corrected separately.

In this study, a threshold adaptive Lambertian–Beckmann model was proposed as Equation (14). Compared with the Lambertian model, this method applies to all common natural targets and provides a comprehensive theory applied to the target from glossy to rough. Correspondingly, an adaptive threshold radiometric calibration method was designed as Equation (15).

The process of radiometric calibration is as follows: (1) Based on the backscatter intensity of each sample at different angles and different wavelengths, the parameters $k_{d}\left(\lambda \right)$ and *m* are calculated by Equation (14) and the least square method. (2) Based on the corresponding parameters in step (1), the backscatter intensity of different angles is corrected according to Equation (15).

**Figure 9:**
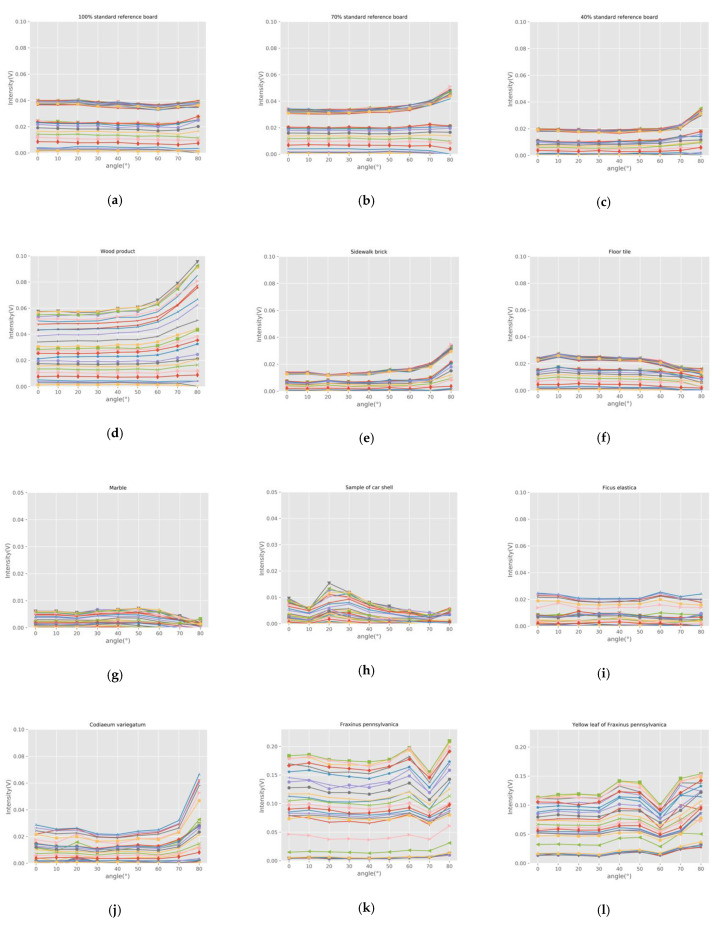

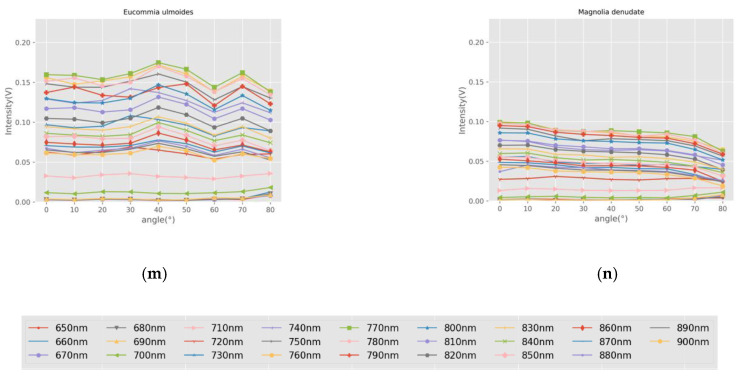
The corrected backscatter intensity of different samples. (**a**) 100% standard reference board; (**b**) 70% standard reference board; (**c**) 40% standard reference board; (**d**) Wood product; (**e**) Sidewalk brick; (**f**) Floor tile; (**g**) Marble; (**h**) Car shell sample; (**i**) Ficus elastic; (**j**) Codiaeum variegatum; (**k**) Fraxinus pennsylvanica; (**l**) Yellow leaf of Fraxinus pennsylvanica; (**m**) Eucommia ulmoides; (**n**) Magnolia denudate.

For the standard reflection boards, sidewalk brick, and wood product in this experiment, the coefficient $k_{d}\left(\lambda \right)$ was close to 1, and the angle threshold $\theta_{T}$ was 0°. We used the second formula in Equation (15) for radiometric calibration. For the samples with obvious specular reflection characteristics, the reflection characteristics were the combination of diffuse reflection and specular reflection. In the process of backscatter intensity radiometric calibration, the influence of diffuse reflection and specular reflection should be considered simultaneously. Based on the fitted model parameters $k_{d}\left(\lambda \right)$ and m, calculated by Equation (14), the backscatter intensities of different angles were calculated by Equation (15). The corrected intensity curves of all samples in this study are shown in Figure 9.

As shown in Figure 6, the original intensity is influenced by the incidence angle. Therefore, the intensity values of the same sample differ significantly, although they have the same scattering property. As shown in Figure 9, the corrected intensity curves of the same sample and same wavelength are approximate. After calibration, the backscatter intensity does not depend on incidence angle and is solely associated with reflectance.

In order to verify the feasibility of the radiometric calibration model proposed in this study, we selected a sample (floor tile) with specular reflection characteristics and a leaf sample (Eucommia ulmoides) as the representative validation samples. We calculated and compared the reflectance of the two samples before and after calibration. The calculation method of reflectance was based on Equation (16).

**Figure 10:**
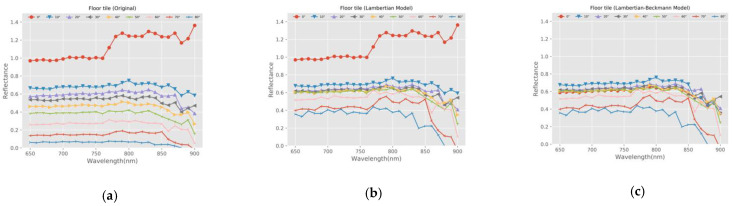
The reflectance comparison of Floor tile. (**a**) Before calibration; (**b**) Calibration results of Lambertian model; (**c**) Calibration results of Lambertian–Beckmann model.

**Figure 11:**
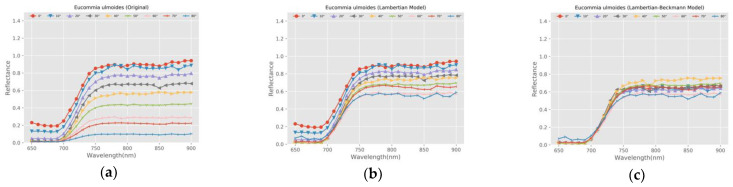
The reflectance comparison of Eucommia ulmoides. (**a**) Before calibration; (**b**) Calibration results of Lambertian model; (**c**) Calibration results of Lambertian–Beckmann model.

Figure 10 and Figure 11 show the reflectance comparison of floor tile and Eucommia ulmoides before and after calibration and the comparison with the calibration results of the Lambertian model.

After analyzing the corrected results, it can be concluded that:

(1) The reflectance of the same target varies greatly at different angles with the effect of incident angle. The reflectance distribution of different angles is discrete before calibration, and the distribution is relatively concentrated after calibration based on the Lambertian model. Compared with the Lambertian model, the Lambertian–Beckmann model is more effective.

(2) For the floor tile with specular reflection characteristics, the Lambertian model is more effective for the calibration of intensity with a larger angle (greater than 20 degrees). The Lambertian–Beckmann model is more effective for the calibration of intensity with 0° and 10°. For the Eucommia ulmoides, the Lambertian model is more effective for the calibration of intensity with angles greater than 30 degrees. The Lambertian–Beckmann model is more effective for the calibration of intensity with 0°, 10°, and 30°. This phenomenon is consistent with the angle threshold of the corresponding sample in Table 2.

(3) The Lambertian–Beckmann model is based on the linear combination of the Lambertian model and the Beckmann model. It combines the calibration advantages of the two models for Lambertian surface and specular surface, respectively, and it is better than the Lambertian model for samples with large specular reflection coefficients, such as floor tile and Eucommia ulmoides. 

All the above results show the feasibility of the proposed radiometric calibration method in a graphical method. To further quantitatively prove the feasibility of this method, we used the mean of the reflectance standard deviations of all wavelengths to measure the effect of calibration. Firstly, we calculated the corrected reflectance based on the Lambertian–Beckmann model of the samples at different incidence angles. The calculation method of reflectance was based on Equation (16). Secondly, we calculated the standard deviation of the reflectance at different incidence angles. Thirdly, we calculated the arithmetic mean of the standard deviations of different wavelengths. Finally, we compared them with that of the results before calibration and after calibration based on the Lambertian model. The results are presented in Table 3.

Furthermore, the size of the laser footprint changes with the incidence angle. With the increase of incident angle, the laser footprint becomes larger so that the energy of the laser return is shared by more points. Therefore, a larger incidence angle may bring new errors and affect the accuracy of the calibration model. In order to verify the accuracy of the calibration model without a large incidence angle, we also calculated the arithmetic mean of the standard deviations of incidence angle less than 70° based on the Lambertian–Beckmann model. The results are also presented in Table 3.

It can be concluded from Table 3 that:

(1) For the samples with diffuse reflection characteristics, the angle threshold, $\theta_{T}$, of the Lambert–Beckmann model is 0°, and the coefficient $k_{d}\left(\lambda \right)$ is close to 1. The Lambert–Beckmann model is equivalent to the Lambertian model. The means of the reflectance standard deviations of all wavelengths before calibration are higher than those of the Lambertian–Beckmann Model (Lambertian Model). The preliminary results suggest that the incident angle effect can be eliminated well by the model, especially for the small incident angles.

(2) For the samples with obvious specular reflection characteristics, such as marble tile and car shell sample, the means of the reflectance standard deviations of the Lambertian model may be equal to or higher than those of the result before calibration. This is because the samples have less diffuse reflection characteristics. The Lambertian model is more suitable for targets with diffuse reflection characteristics, and the calibration results may be worse for the samples with obvious specular reflection characteristics. 

(3) For the samples with $\theta_{T}$ larger than 0°, the results of Lambertian–Beckmann model are better than those of the Lambertian model and the results before calibration. The Lambertian–Beckmann model is effective for the calibration of targets with specular reflection characteristics. The results prove the feasibility of the proposed method.

(4) For the leaf samples in this experiment, the Lambertian–Beckmann model results are better than those of the Lambertian model. The results of the Lambertian model are better than the results before calibration. It can be observed from Figure 7 that the leaves have a stronger specular reflection in the visible wavelength range (650 nm–720 nm) and a stronger diffuse reflection in the near-infrared wavelength range (750 nm–900 nm). The Lambertian model is helpful for the calibration of the angle effect in the near-infrared wavelength range, and the Lambertian–Beckmann model is more helpful for the calibration of the angle effect in the visible wavelength range. It can be observed that Eucommia ulmoides has the lower diffuse reflection coefficient. So, the calibration effect of Eucommia ulmoides is the most obvious.

(5) For all the samples, the arithmetic means of the standard deviations of incidence angle less than 70° based on the Lambertian–Beckmann model are less than those of all angles, apparently. This shows that the error source of the Lambertian model and Lambertian–Beckmann model is partly caused by the larger laser footprint due to a larger incident angle.

## 4. Discussion

The backscatter intensity of HSL represents the light power of the target to the backscatter echo of the emitted laser beam, which cannot be directly used to extract the reflection characteristics of the target. In order to identify the backscatter intensity and characterize the radiation characteristics of the target more realistically, it is necessary to analyze the influence of angle effect on backscatter intensity and study the radiation calibration method.

**Figure 12:**
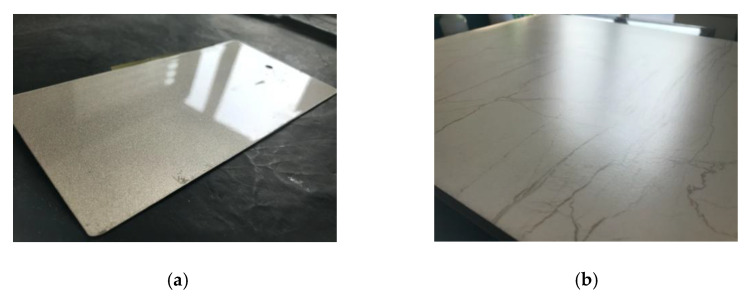
Waxy surfaces, which have ceramic glaze coating outside working as micro-mirrors. (**a**) The car shell sample; (**b**) the floor tile.

In this study, we analyzed the relationship between backscatter intensity and incident angle of HSL and introduced the Lambertian–Beckmann model. A total of 14 kinds of samples were taken into the experiment. The experimental results of different incident angles show that the backscatter intensity recorded by HSL does not fully follow the theoretical Lambertian model. For the targets with isotropic reflection surfaces, such as standard reference boards and the sample of wood product, the relationship between the backscatter intensity and the incident angle has a similar trend, which almost follows the Lambertian model. For the targets with obvious specular reflection, such as the floor tile and the car shell sample, which have ceramic glaze coating outside working as micro-mirrors (Figure 12), there is an obvious specular reflection effect in all measured wavelengths. This result may be helpful to the field of autonomous driving. The specular reflection coefficient of different targets is different, which is related to the characteristics of the target.

For green leaf samples, there is an obvious specular reflection effect near 650–720 nm, which might be related to the inner microstructure of chlorophyll. In the near-infrared wavelengths, the specular reflection effect of the leaf target is low. The green waxy leaves have specular reflection in all of the wavelengths measured and are larger in the visible wavelengths. For the yellow leaf sample, there is almost no specular reflection in the measured wavelengths. Some studies have shown that the reflection of laser from or penetration through a leaf surface and absorption by elements within a leaf depend on the wavelength. At visible wavelengths, chlorophyll absorbs most of the laser that penetrates into a leaf, and so most of the reflected laser is from the leaf surface. At near-infrared wavelengths, the laser is only absorbed by the relatively sparse leaf dry matter, and so there may be significant multiple scattering within the leaf [27]. Therefore, the texture of the leaf surface mainly affects the degree of specular reflection at visible wavelengths. The effect of specular reflection is especially obvious in the leaves with waxy surfaces, and the waxy surface may work in the same way as the glaze coating of the floor tile and car shell samples. At near-infrared wavelengths, there is multiple scattering within the leaf, and so diffuse reflection is the main component. For the yellow leaf, the leaf surface became less shiny and waxy, leading to the decrease of its specular reflection.

**Figure 13:**
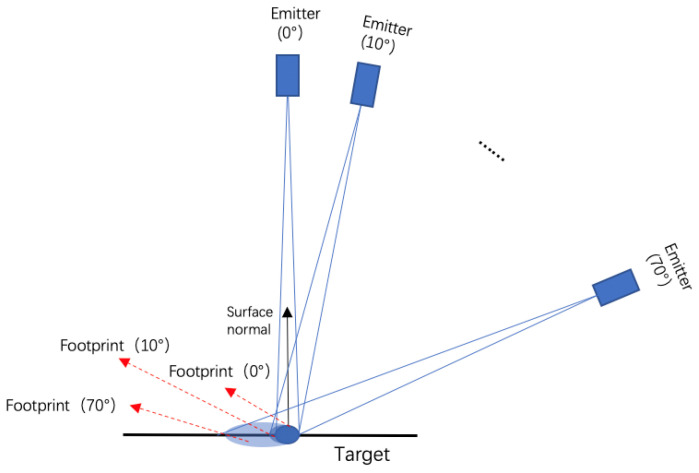
The change of size of laser footprint with incident angle.

In the aspect of calibration method, for the targets with isotropic reflection surfaces, such as standard reference boards and the wood product, the angle threshold of the Lambertian–Beckmann model is 0°, which is almost consistent with the Lambertian model. However, when the incident angle is too large (for example, when the angle is larger than 60°), the calibration error is large. The main reason for this is that the target is not a perfect and uniform diffuse reflector. With the increase of incident angle, the laser footprint becomes larger so that the position of the footprint is different from that of a small incident angle. Therefore, a larger incidence angle may bring new errors and affect the accuracy of the calibration model. The larger the incidence angle is, the larger the measurement error is. When the incident angle is greater than 60°, the size of the laser footprint is about twice or more than twice the size in the normal direction (Figure 13). In this case, the laser footprint is larger than that of the small incident angle so that the energy of the laser return is shared by more points. Therefore, it can be seen from Figure 9 that, when the incident angle is greater than 60°, the calibration effect is obviously worse. In practical applications, the incidence angle of Airborne LiDAR is generally not greater than 60 degrees. Therefore, the calibration method proposed in this paper can be applied to the calibration of the incident angle effect in various application scenarios.

For the samples with obvious specular reflection, there are different degrees of specular reflection effect, and the threshold of incident angle is also different. It can be seen that the smaller the threshold is, the smaller the diffuse reflection coefficient is, and the more obvious the specular reflection effect is. The results of a comparison between the Lambertian model and the Lambertian–Beckmann model show that surface characteristics of leaf samples, the floor tile sample, the marble tile, and the car shell sample do not conform to the Lambertian model. Therefore, the Lambertian model is not suitable for intensity calibration of targets with an obvious specular reflection.

Compared with the Lambertian model, the Lambertian–Beckmann model had higher calibration accuracy, and the maximum improvement rates were 88.97% and 61.90% for the car shell sample and the Eucommia ulmoides sample, respectively. The average improvement rate to the samples in this study was 22.67%. Compared with the results before calibration with the incidence angle less than 70°, the maximum improvement rates of the Lambertian–Beckmann model with the incidence angle less than 70° were 91.65% and 82.90% for the car shell sample and the floor tile sample, respectively. The average improvement rate of the Lambertian–Beckmann model with the incidence angle of less than 70° was 62.26%.

The advantage of the Lambertian–Beckmann model is that the specular reflection effect of the target is considered in the backscatter intensity calibration of HSL, and the diffuse and specular reflection coefficients of the target are calculated by using the backscatter intensity of different angles. A comprehensive theory of intensity calibration is provided, which can be applied to all kinds of surface conditions, from glossy to rough. No matter whether there is a specular reflection effect, the method of this study can be applied to the backscatter intensity calibration. In addition, the Lambertian–Beckmann model is a piecewise function, and the determination of angle threshold greatly affects the calibration accuracy. In this paper, according to the reflection characteristics of different targets and the backscatter intensity of different angles, the angle threshold suitable for the target is calculated to improve the calibration accuracy of backscatter intensity.

However, the proposed method has some limitations. The current research results are based on simple targets and leaves. For more complex scanning scenes and trees, the incident angle is usually difficult to obtain. Further research is needed to study the influence of multiple scattering. In addition, the surface roughness or grain size of different targets are different. For targets with no specular reflection and a great influence on roughness, their reflection characteristics do not necessarily follow the Lambertian model. For the target without specular effect, it is necessary to further study the calibration model of angle effect under the influence of roughness and other factors.

Based on the model proposed in this paper, the angle threshold and the specular reflection component are calculated adaptively. The backscatter intensity of different angles of incidence is corrected to the normal direction of incidence, and the reflectance is calculated. The results can be used for further target classification and to improve classification accuracy.

## 5. Conclusions

This study presented a new method to eliminate the incident angle effect of HSL. The relationship between backscatter intensity and incident angle of HSL was studied by combining theory with experiments, and an adaptive threshold incident angle effect calibration model was proposed. Based on the linear combination of the Lambertian model and the Beckmann model, we provided a comprehensive theory that can be applied to all kinds of surfaces, from glossy to rough. It can be used to correct the measured backscatter intensity of various incident angles, and it is no longer affected by the incidence angle and is only related to the target reflectance. By considering the effects of diffuse reflections and specular reflections, the incidence angle effect can be accurately corrected. Compared with the Lambertian model, the proposed model had higher calibration accuracy, and the maximum two improvement rates were 88.97% and 61.90% for two samples in the experiment, respectively. The average improvement rate to the samples in this study was 22.67%. Compared with the results before calibration with the incidence angle of less than 70°, the average improvement rate of Lambertian–Beckmann model with the incidence angle of less than 70° was 62.26%. It can be concluded from the calibration results that the Lambertian–Beckmann model is more accurate than the Lambertian model. This is a meaningful and breakthrough exploration for HSL.

In general, natural surfaces are very complex and can be simulated as a combination of specular reflection and diffuse reflection. The Lambertian–Beckmann model successfully quantifies the difference between diffuse and specular reflectance coefficients. Compared with the Lambertian model, this method is suitable for most natural surfaces at different incident angles. For surfaces with none or small specular reflection, the Beckmann model becomes the Lambertian model as the incident angle threshold is close to 0°. On the contrary, for the glossy surface with large specular reflection, the method proposed in this paper can estimate the diffuse and specular reflection coefficients of different targets and calculate the incident angle threshold at the same time. In our daily life, there are many artificial targets with ceramic glaze coating or waxy material outside working as micro-mirrors. The Lambertian–Beckmann model is helpful for more targets classification and may be helpful in the field of autonomous driving. Moreover, we also found that the green leaves have an obvious specular reflection effect near to 650–720 nm, which might be related to the inner microstructure of chlorophyll. The green leaves have a stronger diffuse reflection in the near-infrared wavelength range. The Lambertian–Beckmann model is helpful for the calibration of angle effect in all wavelength ranges of green leaves. It lays the foundation for more meaningful work, such as target classification based on HSL point cloud measured in varying incident angles, and considerably mitigates the problems caused by the angle effect.

However, in the scanning process of HSL, the backscatter intensity is affected by many factors, such as the characteristics of the instrument system, the distance, the environment, and so on. In order to obtain accurate backscatter intensity information and correctly evaluate the response of targets to laser pulses, it is necessary to remove the influence of various factors. In the future, we need to further study the influence of HSL instrument system characteristics, the distance effect, the environment, and other factors, so as to provide the necessary data basis for the better quantitative application of HSL.

## Figures and Tables

**Table 1:** Experimental samples and corresponding photos.

Samples	Photos	Samples	Photos
99% standard reference board	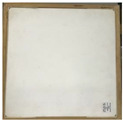	70% standard reference board	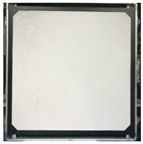
40% standard reference board	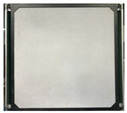	Wood product	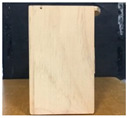
Sidewalk brick	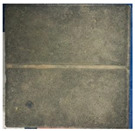	Floor tile	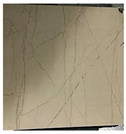
Marble tile	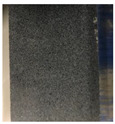	A car shell sample	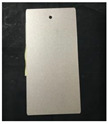
Fraxinus pennsylvanica	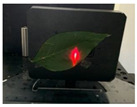	Yellow leaf of Fraxinus pennsylvanica	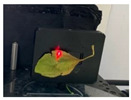
Eucommia ulmoides	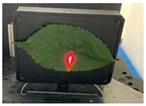	Magnolia denudate	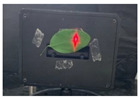
Codiaeum variegatum	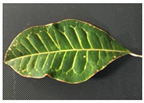	Ficus elastic	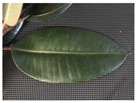

**Table 2:** The relationship between $\theta_{T}$ and different wavelength.

Wavelength (nm)	$\theta_{T}\;({}^{\circ})$							
	Floor Tile	Car Shell Sample	Marble Tile	Fraxinus Pennsylvanica	Eucommia Ulmoides	Magnolia Denudate	Ficus Elastic	Codiaeum Variegatum
650	10	30	10	20	20	20	30	20
660	10	30	10	20	20	30	30	30
670	10	30	10	30	20	30	20	20
680	10	30	10	30	20	30	20	20
690	10	30	10	20	20	30	20	30
700	10	30	10	20	20	20	20	20
710	10	30	10	0	20	20	10	20
720	10	30	10	0	20	20	0	10
730	10	30	10	0	20	0	0	0
740	10	30	10	0	20	0	0	0
750	10	30	20	0	20	0	0	0
760	10	30	10	0	20	0	0	0
770	10	30	20	0	20	0	20	0
780	10	30	20	0	30	0	30	0
790	10	30	20	0	30	0	20	0
800	10	30	20	0	30	0	30	0
810	10	30	10	0	30	0	20	0
820	10	30	10	0	30	0	20	0
830	10	30	20	0	30	0	40	0
840	10	30	20	0	30	0	30	10
850	10	30	20	0	20	0	30	10
860	20	30	10	0	30	0	30	10
870	20	30	10	0	20	0	50	20
880	20	30	10	0	20	10	40	20
890	20	30	10	0	20	0	40	20
900	20	20	30	0	30	0	30	10

**Table 3:** The mean of the reflectance standard deviations of all wavelengths.

Samples	Calibration Type	The Mean of the Reflectance Standard Deviations of All Wavelengths
70% standard reference board	Before calibration	0.244
	Lambertian Model	0.119
	Lambertian–Beckmann Model	0.119
	Lambertian–Beckmann Model (with incidence angle less than 70°)	0.035
40% standard reference board	Before calibration	0.122
	Lambertian Model	0.108
	Lambertian–Beckmann Model	0.108
	Lambertian–Beckmann Model (with incidence angle less than 70°)	0.023
Wood product	Before calibration	0.216
	Lambertian Model	0.134
	Lambertian–Beckmann Model	0.134
	Lambertian–Beckmann Model (with incidence angle less than 70°)	0.037
Sidewalk brick	Before calibration	0.171
	Lambertian Model	0.153
	Lambertian–Beckmann Model	0.153
	Lambertian–Beckmann Model (with incidence angle less than 70°)	0.040
Floor tile	Before calibration	0.326
	Lambertian Model	0.24
	Lambertian–Beckmann Model	0.128
	Lambertian–Beckmann Model (with incidence angle less than 70°)	0.0486
Marble tile	Before calibration	0.052
	Lambertian Model	0.058
	Lambertian–Beckmann Model	0.034
	Lambertian–Beckmann Model (with incidence angle less than 70°)	0.015
Car shell sample	Before calibration	0.562
	Lambertian Model	0.562
	Lambertian–Beckmann Model	0.062
	Lambertian–Beckmann Model (with incidence angle less than 70°)	0.051
Fraxinus pennsylvanica	Before calibration	0.166
	Lambertian Model	0.05
	Lambertian–Beckmann Model	0.048
	Lambertian–Beckmann Model (with incidence angle less than 70°)	0.028
Yellow leaf of Fraxinus pennsylvanica	Before calibration	0.11
	Lambertian Model	0.075
	Lambertian–Beckmann Model	0.075
	Lambertian–Beckmann Model (with incidence angle less than 70°)	0.043
Eucommia ulmoides	Before calibration	0.222
	Lambertian Model	0.105
	Lambertian–Beckmann Model	0.04
	Lambertian–Beckmann Model (with incidence angle less than 70°)	0.035
Magnolia denudate	Before calibration	0.1
	Lambertian Model	0.043
	Lambertian–Beckmann Model	0.04
	Lambertian–Beckmann Model (with incidence angle less than 70°)	0.022
Ficus elastic	Before calibration	0.141
	Lambertian Model	0.098
	Lambertian–Beckmann Model	0.059
	Lambertian–Beckmann Model (with incidence angle less than 70°)	0.046
Codiaeum variegatum	Before calibration	0.227
	Lambertian Model	0.185
	Lambertian–Beckmann Model	0.176
	Lambertian–Beckmann Model (with incidence angle less than 70°)	0.048

## Data Availability

Not applicable.
